# Warm Acclimation Modulates the Physiological Responses of Young *Saccharina japonica* to Marine Heatwaves

**DOI:** 10.3390/biology15161398

**Published:** 2026-08-14

**Authors:** Yuning Xue, Yue Wang, Dong Xu, Xiaodong Li, Xinhua Chen, Yaoyao Chu, Xiongwei Huang

**Affiliations:** 1 State Key Laboratory of Mariculture Breeding, Key Laboratory of Marine Biotechnology of Fujian Province, College of Marine Sciences, Fujian Agriculture and Forestry University, Fuzhou 350002, China; 52425014007@fafu.edu.cn (Y.X.); 52525014046@fafu.edu.cn (Y.W.); lixiaodong11@163.com (X.L.); chenxinhua@tio.org.cn (X.C.); 2 State Key Laboratory of Mariculture Biobreeding and Sustainable Goods, Yellow Sea Fisheries Research Institute, Chinese Academy of Fishery Sciences, Qingdao 266071, China; xudong@ysfri.ac.cn

**Keywords:** commercial seaweed, marine heatwaves, growth, photosynthetic apparatus, *Saccharina japonica*

## Abstract

Frequent marine heatwaves (MHWs) pose significant threats to and have a profound impact on the structure and functioning of marine ecosystems. The response of marine organisms to MHWs may depend on the background temperature of their habitats. *Saccharina japonica* is a cold-temperate species that is vulnerable to thermal stress. However, it remains unclear how habitat-related temperatures impact responses of *S. japonica* to MHWs. In this study, the algae were exposed to MHWs (+∆4 °C) under two background temperatures (control level, 8 °C; warmer level, 12 °C), and the growth, photosynthetic performance, and biochemical composition were measured at the end of the MHW and the recovery periods. The results showed that a warmer background temperature increased the growth and pigment contents (Chlorophyll *c* and fucoxanthin) of *S. japonica*, but decreased chlorophyll fluorescence parameters, including the effective quantum yield, relative electron transport rate, non-photochemical quenching, and photochemical quenching. The MHWs had no significant effect on growth rate, photosynthetic performance, or biochemical compositions at the background temperature of 8 °C. In contrast, MHWs reduced growth and biochemical composition contents (Chlorophyll *a*, Chlorophyll *c*, fucoxanthin and soluble protein) at a warmer level, but the negative influence was alleviated after the recovery period. Furthermore, there was a considerable increase in the total antioxidative capacity caused by MHWs under warmer conditions. Overall, the stimulation in growth demonstrated that warmer conditions (12 °C) may provide a better growth temperature for young *S. japonica* but also increase the risks of biomass losses and physiological damage triggered by MHWs. These findings deepen our understandings of the tolerance and resistance of *S. japonica* to heatwaves and provide useful information for managing and modulating the cultivation of this important commercial seaweed.

## Simple Summary

In coastal ecosystems, macroalgae serve as fundamental primary producers that sustain biotic diversity, stabilize community structure, and maintain integral coastal ecological functions. *Saccharina japonica* is one of the most extensively cultivated brown macroalga along the Chinese coastal regions. At present, frequent marine heatwaves (MHWs) threaten the productivity and quality of this macroalga. Furthermore, the MHWs-resilience of seaweed may be affected by their growth background temperatures. This study examined the effect of MHWs on the growth and physiological traits of young *S. japonica* under two initial acclimation temperatures. The results revealed that warmer conditions beneficially influenced the growth and biochemical composition of young *S. japonica*, but suppressed photosystem II function. MHWs had no significant effect on the growth rate, photosynthetic performance, or biochemical compositions at control temperatures. In contrast, MHWs had a negative influence on growth and photosynthetic pigment concentration in the warmer treatment, but the negative influence was alleviated after a recovery period. Overall, these findings suggested that warmer local conditions may improve young *S. japonica* growth, but would also increase the potential risk posed by MHWs to this species’ biomass and quality.

## 1. Introduction

In coastal ecosystems, macroalgae serve as fundamental primary producers that sustain biotic diversity, stabilize community structure, and maintain integral coastal ecological functions [1,2]. As benthic sessile organisms that complete their entire life cycle within fixed habitats, macroalgae rely on metabolic plasticity to acclimate to environmental fluctuations and mitigate stress-induced impairments [3,4,5]. Among many environmental fluctuations, rising seawater temperature is thought to be the primary threat to macroalgae [6,7]. Each macroalgal species possesses a specific thermal optimum; even subtle temperature elevations beyond their critical physiological thresholds can trigger pervasive detrimental impacts on growth or metabolism, and may ultimately result in population decline or mass mortality [8,9]. For example, many habitat-forming kelps and fucoid exposed to acute or chronic thermal stress show heat-induced decreases in chlorophyll content and photosynthetic efficiency, indicating photosynthetic apparatus damage [10,11,12,13]. Thus, clarifying the influence of high temperatures on macroalgae is critical for the protection and management of macroalgal resources.

Similar to natural seaweed assemblages, artificial seaweed aquaculture beds can reconstruct simplified coastal microecosystems and provide a variety of ecological benefits to associated organisms [14,15,16]. Compared to natural macroalgal populations, cultured seaweeds achieve better growth conditions by selecting suitable farming sites. However, the instability of farming area conditions and the potential consequences of fluctuating marine environments on seaweed cultivation are currently becoming increasingly evident due to ongoing global climate changes [17,18]. Among climate-driven environmental perturbations, thermal stress has been identified as the dominant limiting factor for seaweed yield and productivity [19,20,21,22]. Marine heatwaves (MHWs), as extremely high-temperature events, exert severe detrimental impacts on commercially valuable macroalgal species and are projected to amplify thermal stress pressure under future warming scenarios [23,24,25]. In the last decade, MHWs in China seas have shown significant increasing trends in terms of intensity and frequency. Specifically, this has become more common in coastal seas than in open seas [26]. Severe MHWs could reach above 3 °C in winter [27]. Consequently, extreme high-temperature events constitute a serious threat to the long-term growth of macroalgae aquaculture, and the impacts of MHWs on seaweed farming should be investigated further.

*Saccharina japonica* is one of the most extensively cultivated brown macroalga along the Chinese coastal regions [28,29]. In 2025, the production of *S. japonica* in China reached over 180 × 10^4^ t, accounting for almost 62% of cultured seaweed production [30]. As a typical cold-temperate macroalgal species, *S. japonica* is inherently vulnerable to thermal perturbation [31]. Elevated seawater temperatures can profoundly disrupt core physiological and biochemical processes, including photosynthesis performance and biomass generation [32,33]. Additionally, young *S. japonica* sporophytes are routinely transplanted from indoor nursery facilities to coastal waters. Unfavorable aquatic temperature might cause crop loss if kelp is cultivated too early in the field to elongate the cultivation period [17]. Previous studies have defined the optimal growth temperature range for cultivated *S. japonica* as 5–10 °C [34]. However, growth declined as seawater temperature increased, and even a 1 °C increase can significantly limit kelp production [33,35]. Accordingly, an appropriate aquatic temperature during the sporophyte transplantation stage is critical for successful outdoor cultivation and stable yield maintenance of *S. japonica*. In addition to constant high seawater temperature stress, more frequent and stronger MHWs have become a new thermal challenge for *S. japonica* production. Accumulating evidence confirms that prior thermal acclimation history profoundly modulates macroalgal responses to subsequent acute thermal stress [36], implying that algal MHW resilience varies as a result of adapting to different local seawater temperatures [37,38,39]. However, the effect of growth background temperature on the resilience of *S. japonica* to MHWs is still unknown.

Early developmental stages of macroalgae are generally most vulnerable to ambient environmental stress [40,41], emphasizing the necessity of evaluating thermal responses in young *S. japonica* sporophytes under variable thermal regimes. Regional climate assessments have reported that the intensity of severe MHWs was more than 3 °C in the China Sea [27], and ongoing anthropogenic climate change is projected to further amplify the magnitude, frequency, and severity of MHW events in coastal oceans [42], posing more severe thermal threats to coastal macroalgal aquaculture. Therefore, in order to investigate the resilience of young *S. japonica* to MHWs and assess growth background temperature effects on the algae response to acute thermal stress, a combined experiment between an MHW intensity of +∆4 °C and two growth background temperatures (control level, 8 °C; warmer level, 12 °C) was conducted in this study. The growth rates, photosynthetic performance, and biochemical compositions of this alga were examined under various temperature scenarios. The findings will provide practical information for analyzing the suitable cultivation temperature for kelp farming when confronted with extreme elevated temperatures during cultivation in coastal waters.

## 2. Materials and Methods

### 2.1. Sample Collection and Preculture Conditions

In January 2026, healthy young thalli of *S. japonica* with a length of approximately 50 cm were collected from farming areas of Xiapu (119.96° E, 26.59° N) in Ningde, China. The samples were transported to the laboratory in a cooling box within 3 h of collection. They were then rinsed repeatedly with filtered seawater to remove sediment, epiphytes, and surface contaminants. Then, the discs (diameter = 1.2 cm) were excised from the meristematic area of thalli and placed in glass flasks with filtered natural seawater, enriched with 50 µM NO_3_^−^ and 4 µM PO_4_^3−^. The cultivation condition was based on studies referring to a previous study on *S. japonica* [43], and the supplemented nutrient level was similar to the seawater in terms of nitrogen and phosphorus concentrations. Every flask was continuously aerated. Seawater was renewed every day to supply enough nutrients. The temperature was monitored daily and maintained at 8 ± 0.5 °C. The light intensity was set at 90 µmol photons m^−2^ s^−1^ with a 12 h:12 h light and dark cycle. Before beginning experimental treatments, the algae were acclimated for three days under these circumstances to allow healing of marginal wounds.

### 2.2. Experimental Setup and Algae Culture Conditions

**Figure 1:**
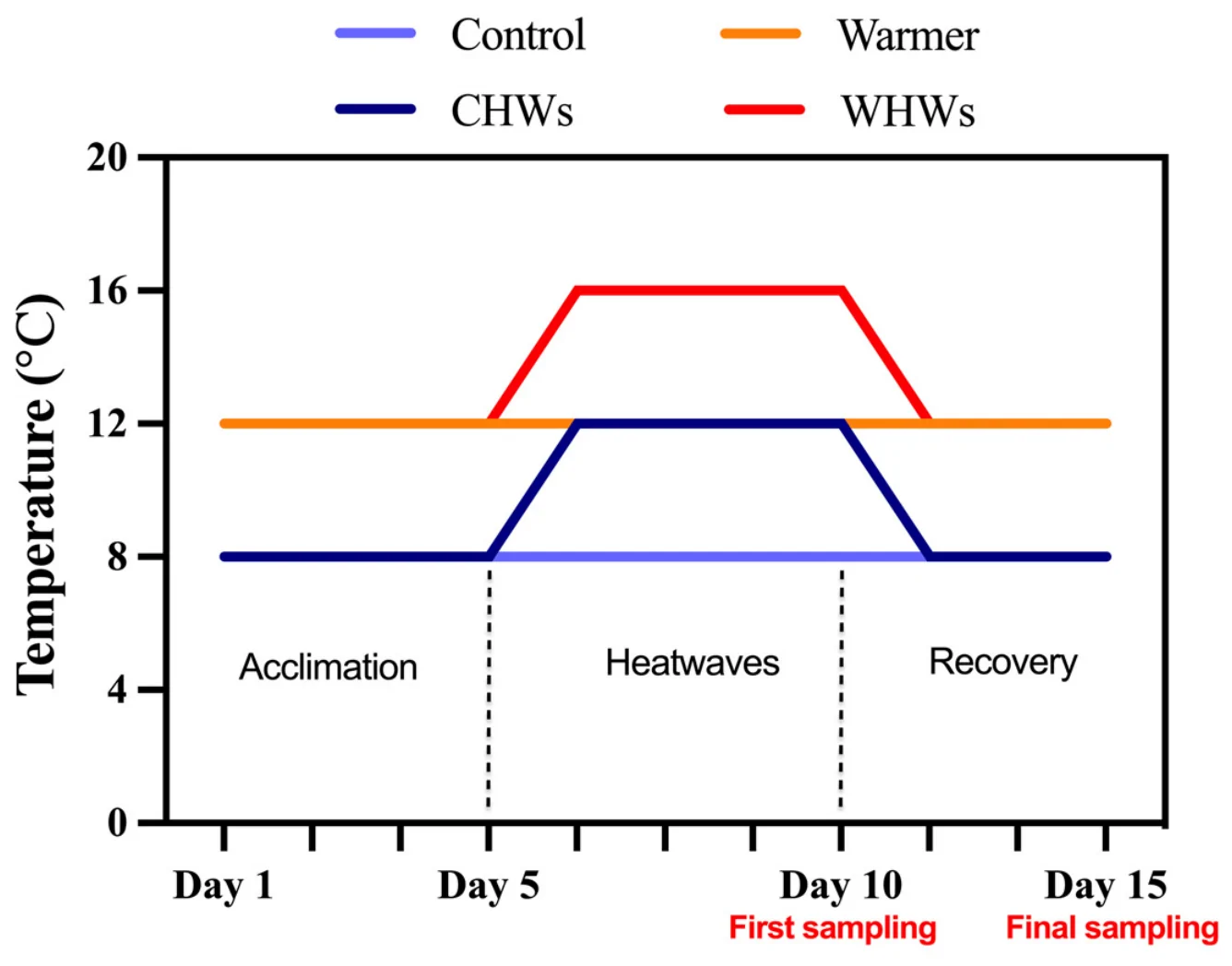
Experimental timeline of the sampling regime and temperature development of the 4 temperature scenarios (Control, 8 °C; Control + ∆4 °C, CHWs; Warmer, 12 °C; Warmer + ∆4 °C, WHWs) during acclimation, heatwave, and recovery periods. The experiments lasted 15 days in total. The first sampling and final sampling was undertaken at the end of the heatwave and the recovery period on day 10 and 15, respectively.

Following the precultured period, the kelp discs were randomly assigned to various temperature scenarios. At the beginning, the discs were independently cultivated at two background temperatures (8 °C and 12 °C) for five days (Figure 1, Acclimation) with 10 flask replicates. Six discs were put into each flask, which was then gently aerated. Following acclimation, five replicates for each background temperature treatment were maintained constantly at either 8 °C or 12 °C throughout the experiment. Another five replicates per group experienced a 5-day heatwave (+Δ4 °C), followed by a 5-day recovery phase (Figure 1, Heatwaves and Recovery). Temperatures were ramped up at 1 °C h^−1^ to the target heatwave temperature, then reduced at 1 °C h^−1^ back to their original temperatures (8 °C or 12 °C) upon heatwave termination (Figure 1, Recovery). Therefore, the experiments included four treatments, namely, growth background temperature of 8 °C (collection sites temperature, control), heatwaves at a growth background temperature of 8 °C (CHWs), growth background temperature of 12 °C (warmer), and heatwaves at a growth background temperature of 12 °C (WHWs). To quantitatively compare thermal injury and recovery potential under different background temperatures while minimizing confounding temporal effects, equal durations were assigned for heatwave exposure and recovery, as reported in prior studies [44,45,46,47].

For each replicate, 1.5 g discs were maintained in 1 L of filtered natural seawater supplemented with NaNO_3_ and Na_2_HPO_4_, with final concentrations of 100 µM for NO_3_^−^ and 15 µM for PO_4_^3^^−^. Nutrient limiting did not occur at the applied nutrient level during the experiment based on a preliminary experiment. The light intensity and photoperiod were kept constant, in line with preculture conditions. The medium was replenished every 3 days. The growth, photosynthetic traits, photosynthesis pigments, soluble protein content, and total antioxidative capacity (T-AOC) were assessed at the end of the heatwave exposure (Figure 1, first sampling on day 10) and after recovery (Figure 1, final sampling on day 15).

### 2.3. Relative Growth Rate

The relative growth rates (RGR) of *S. japonica* were determined by measuring the fresh weight of all discs in each flask at the start and at a specific time point during the experiment. The surface water of the samples was removed using paper towels until no more changes in biomass were detected. RGR was calculated as follows:

RGR (% day^−1^) = (ln W_t_ − ln W_0_)/t × 100 (1)

where W_0_ and W_t_ represent the initial fresh weight and the fresh weight at time t, respectively.

### 2.4. Chlorophyll Fluorescence Parameters

Chlorophyll fluorescence characteristics were determined using the Maxi version of an IMAGING-PAM (M-Series, Walz, Effeltrich, Germany). The maximum photochemical quantum yield (F_v_/F_m_) of photosystem II (PSII) was determined using the equation: F_v_/F_m_ = (F_m_ − F_0_)/F_m_. F_0_ represents the minimum fluorescence after dark adaptation, measured under low measuring light intensity, and F_m_ was obtained with a saturation light pulse of 4000 µmol photons m^−2^ s^−1^. Following 15 min of dark acclimation, the relative electron transport rate (rETR), and non-photochemical quenching (NPQ) were directly evaluated under the culture light intensity. Rapid light curves (RLCs) were determined at ten progressively rising actinic light levels (0, 1, 11, 21, 36, 56, 81, 111, 146, 186 µmol photons m^−2^ s^−1^) at 20 s intervals. The maximum rETR (rETR_max_) was obtained using non-linear fitting of the rapid light curves. The RLC was fitted applying the equation by Webb et al. (1974) [48]:

rETR = rETR_max_ (1 − e^−αI/rETRmax^) (2)

### 2.5. Photosynthetic Pigments

Pigments were measured using the modified method of Seely et al. (1972) [49]. A total of 1 mL of dimethyl sulfoxide (DMSO) and about 0.1 g of fresh tissue was added to a tube, which was then left in the dark for 15 min. The absorbance of the supernatant was measured at 480, 582, 631, and 665 nm. The following formulas were used to determine the concentration of chlorophyll *a* (Chl *a*), chlorophyll *c* (Chl *c*), and fucoxanthin (F_x_):

Chl *a* = A_665_/72.8 (3)

Chl *c* = (A_631_ + A_582_ − 0.297A_665_)/61.8 (4)

F_x_ = (A_480_ − 0.772 (A_631_ + A_582_ − 0.287A_665_) − 0.049A_665_)/130 (5)

### 2.6. Soluble Protein (SP) and Total Antioxidative Capacity (T-AOC)

Algal samples (0.1 g) were crushed in liquid nitrogen before being transferred into 0.9 mL of phosphate buffer (0.1 mol L^−1^, pH 7.0). After centrifuging at 2500 rpm for 10 min, the supernatant was collected in order to measure the soluble protein (SP) and T-AOC. The total amount of SP was determined by the Bradford method (1976) [50] using bovine serum albumin as standard. The 1 mL of supernatant was added with 9 mL of normal saline. The resulting pellets were treated with 3 mL of Coomassie Brilliant Blue reagent. After a reaction of 10 min, the absorbance of the mixture supernatant was measured at 595 nm. T-AOC was estimated using commercial assay kits (Nanjing Jiancheng Bioengineering Institute, Nanjing, China), in accordance with the manufacture’s instructions. T-AOC was measured with a colorimetric kit (Nanjing Jiancheng, China). Antioxidants in algal homogenates reduce Fe^3+^ to Fe^2+^, which forms stable chromogenic complexes with phenanthroline reagents. Absorbance was read at 520 nm to calculate total antioxidant capacity.

### 2.7. Statistical Analyses

The statistical analyses in this study are provided as mean values ± standard deviations (means ± SDs) and completed using SPSS 25.0. Data from each treatment were found to have a normal distribution (Shapiro–Wilk test, *p* > 0.05) and homogeneous variances (Levene’s test, *p* > 0.05). A One-way analysis of variance (ANOVA) was used to assess the effects of temperature scenarios (fixed factor) on all measured parameters, including growth, chlorophyll fluorescence characteristics, photosynthetic pigments, SP contents, and T-AOC. Significant differences among treatment groups were investigated further using Duncan’s post hoc test. For normally distributed data, *p* < 0.05 was used as the level of significance.

## 3. Results

### 3.1. Relative Growth Rates

**Figure 2:**
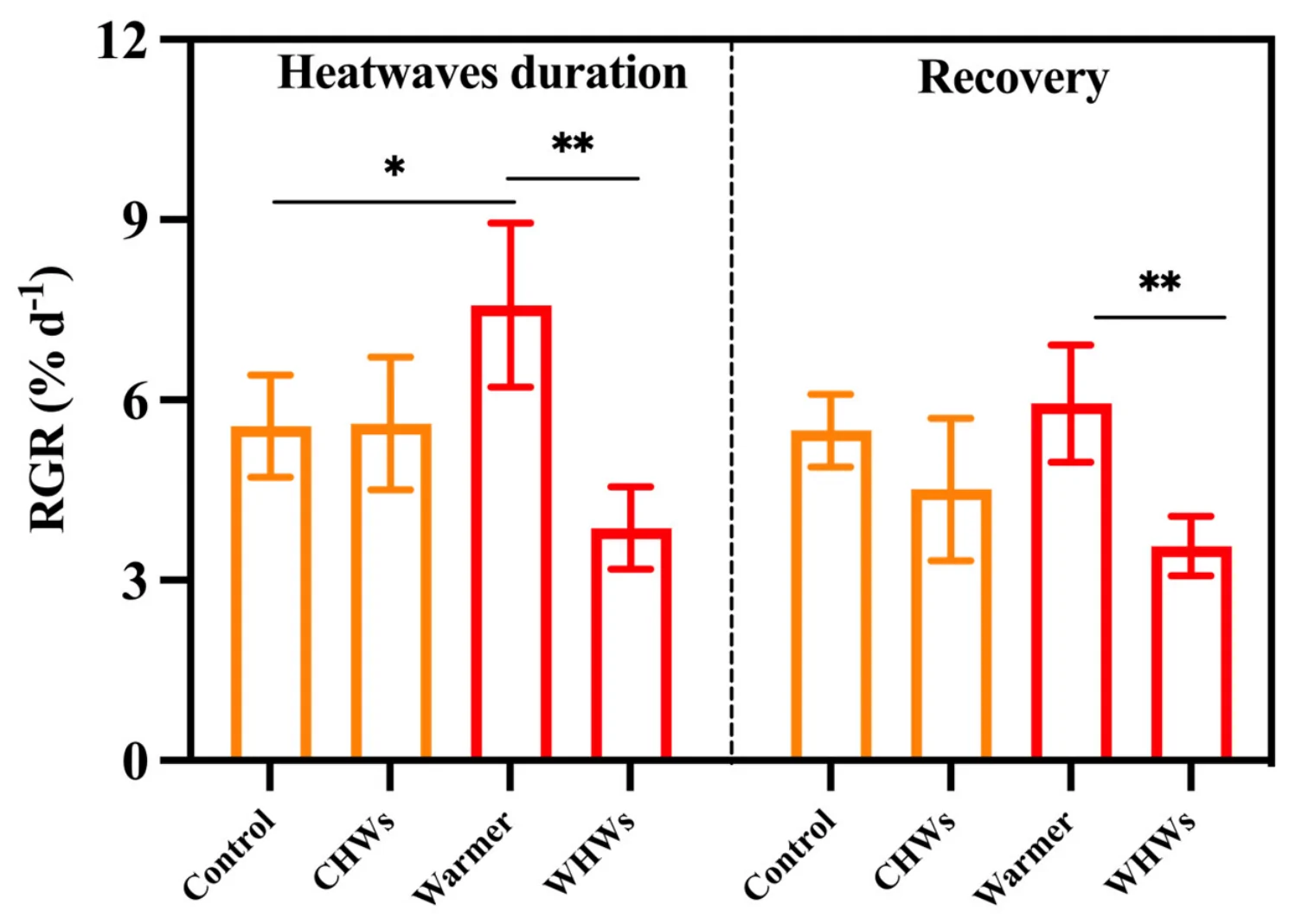
The relative growth rate (RGR) of young *Saccharina japonica* cultured under four temperature scenarios. Data represent mean ± SD (n = 5). Statistical significance was defined as *p* < 0.05 (*) and *p* < 0.01 (**).

At the end of the heatwave period, no significant differences in RGR were observed between the control and CHW treatments (Figure 2). Contrarily, a significant difference was detected between warmer and WHW treatments. The RGR under WHW conditions was 49% lower than that under warmer conditions. Compared to the control treatment, the RGR under warmer conditions was significantly enhanced, by 36% (Figure 2). By contrast, the RGR under the WHW treatment was inhibited by 31% compared to that under the CHW treatments (Figure 2).

At the end of the recovery phases, there were no significant differences in RGR between the control and CHW treatments. In contrast, under WHW treatments, RGR was significantly lowered, by 40%, than that under the warmer treatment (Figure 2). However, there were no significant differences between the warmer and control treatments (Figure 2). Similarly, the RGR under WHW treatment showed no significant difference compared to that under CHW (Figure 2).

### 3.2. Chlorophyll a Fluorescence

**Figure 3:**
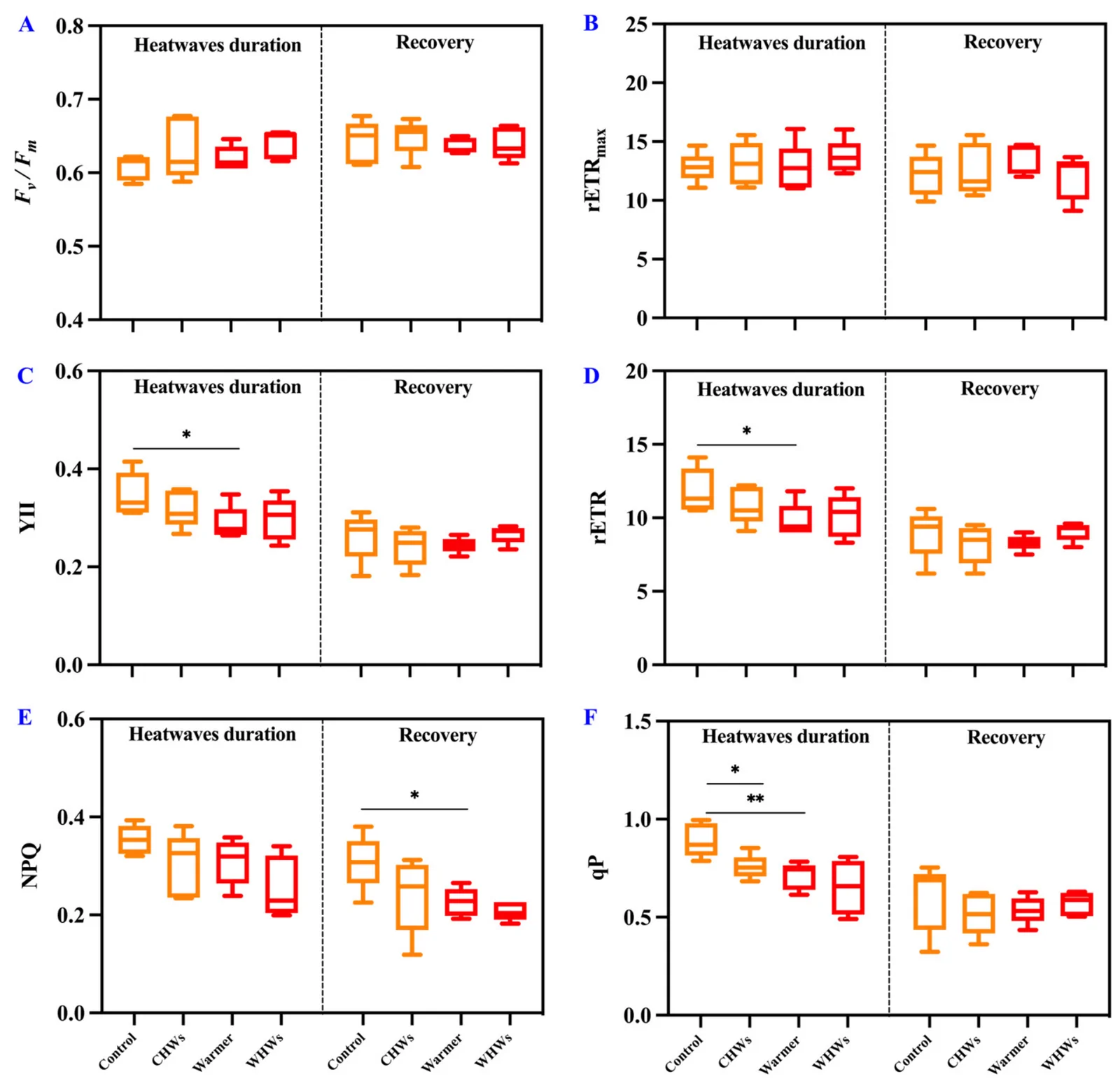
The maximum photochemical quantum yield of PSII (F_v_/F_m_; (**A**)), maximum rETR (rETR_max_; (**B**)), effective photochemical quantum yield (YII; (**C**)), relative electron transport rate (rETR; (**D**)), non-photochemical quenching (NPQ; (**E**), and photochemical quenching (qP; (**F**)) of young *Saccharina japonica* cultured under four temperature scenarios. Data represent mean ± SD (n = 5). Statistical significance was defined as *p* < 0.05 (*) and *p* < 0.01 (**).

At the end of the heatwave period, the values of F_v_/F_m_, rETR_max_, and NPQ showed no significant difference among the four temperature scenarios (Figure 3A–F). In contrast, the YII, rETR, and qP under warmer treatments were significantly inhibited—by 17%, 17%, and 20%, respectively—compared to the control treatment (Figure 3C,D,F). In addition, under the CHW treatment, qP was significantly decreased, by 15%, relative to the control treatment (Figure 3F).

Furthermore, at the end of recovery period, a significant difference was found only in the NPQ values. There was a 48% reduction under the warmer treatment compared to the control treatment (Figure 3E). No significant difference in the values of F_v_/F_m_, rETR_max_, YII, rETR, or qP were detected across the four temperature treatments (Figure 3A–D,F).

### 3.3. Chlorophyll and Fucoxanthin Contents

**Figure 4:**
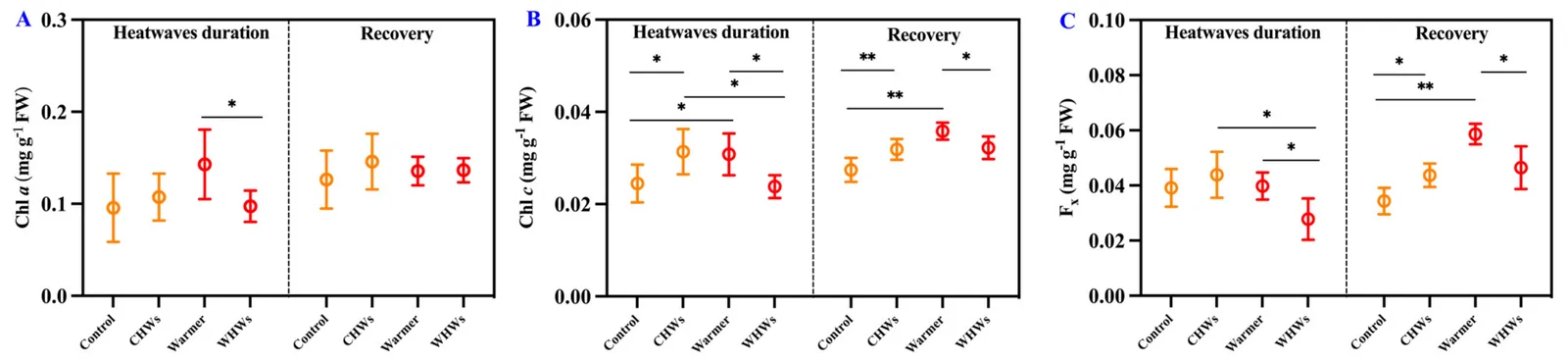
The Chl *a* (**A**), Chl *c* (**B**) and F_x_ (**C**) contents of young *Saccharina japonica* cultured under four temperature scenarios. Data represent mean ± SD (n = 5). Statistical significance was defined as *p* < 0.05 (*) and *p* < 0.01 (**).

At the end of the heatwave period, the Chl *c* content was significantly enhanced by CHWs relative to the control treatment, with a 28% increase, while the Chl *a* and F_x_ contents showed no significant difference between the control and CHW treatments (Figure 4B,C). In contrast, the Chl *a*, Chl *c* and F_x_ contents were significantly inhibited by heatwaves under warmer conditions, with a reduction of 32%, 23%, and 30%, respectively (Figure 4A–C). By contrast, compared to the control, a 26% increase was found in Chl *c* under the warmer treatment (Figure 4B), while no significant changes were found in the Chl *a* and F_x_ contents (Figure 4A,C). Meanwhile, compared to CHWs, the Chl *c* and F_x_ contents under WHW treatment were significantly decreased–by 24% and 37% (Figure 4B,C), respectively–whereas no significant changes were observed in the Chl *a* content (Figure 4A).

At the end of the recovery period, there were no significant differences in Chl *a* content among the four temperature scenarios (Figure 4A–C). Meanwhile, the heatwaves and warmer conditions induced a change in Chl *c* and F_x_ content. Compared to the control, the CHWs significantly increased Chl *c* and F_x_, by 16% and 27% (Figure 4B,C), respectively. On the contrary, compared to the warmer treatment, the WHWs induced a significant decrease in Chl *c* and F_x_, by 10% and 21% (Figure 4B,C), respectively. Additionally, compared to the control treatment, a significant increase in Chl *c* and F_x_ was found under warmer treatments, of 30% and 71%, respectively (Figure 4B,C).

### 3.4. Soluble Protein and Total Antioxidative Capacity

**Figure 5:**
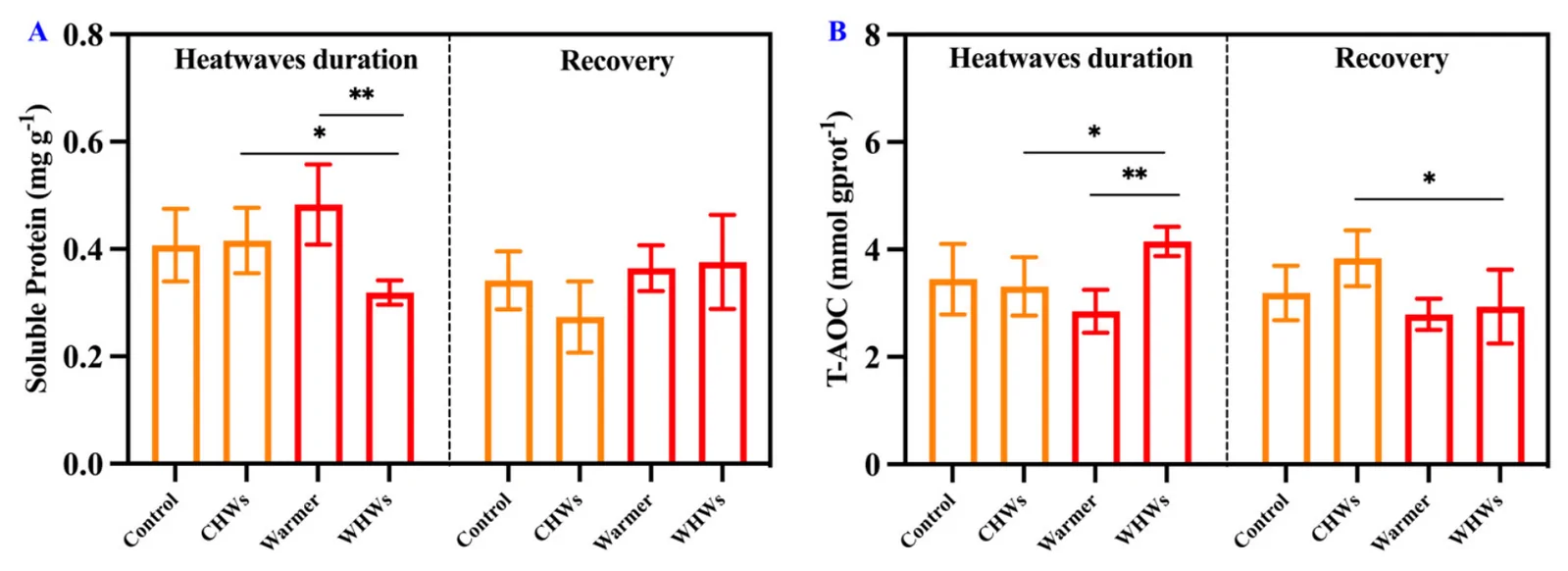
The soluble protein (SP; (**A**)) and total antioxidative capacity (T-AOC; (**B**)) of young *Saccharina japonica* cultured under four temperature scenarios. Data represent mean ± SD (n = 5). Statistical significance was defined as *p* < 0.05 (*) and *p* < 0.01 (**).

At the end of the heatwave period, neither SP nor T-AOC showed any significantly difference between the control and CHW treatments (Figure 5A,B), while the SP and T-AOC were significantly inhibited, increasing by 34% and 45% under WHW conditions compared to the warmer treatment (Figure 5A,B), respectively. Furthermore, the SP content was significant inhibited under the WHW treatment, with a 23% reduction compared to the CHW treatment (Figure 5A). Meanwhile, T-AOC was significantly increased, by 25%, under WHW treatment compared to CHW treatment (Figure 5B).

At the end of the recovery period, the SP showed no significant difference among the four temperature scenarios (Figure 5A). In contrast, T-AOC was significantly decreased under WHW treatment, showing a reduction of 24% compared to the CHW treatment (Figure 5B).

## 4. Discussion

### 4.1. Growth Resilience of Young S. japonica to MHWs Under Different Background Temperatures

The effects of heatwaves on young *S. japonica* sporophytes were studied at two background seawater temperatures. At the control condition (8 °C), heatwaves with an intensity of +∆4 °C induced no significant alterations in growth of the kelp. In contrast, warmer conditions of 12 °C markedly promoted the growth performance of sporophytes. Consistent with our results, Gao et al. (2017) [51] reported growth stimulation in *S. japonica* when seawater temperatures rose from 5 °C to 10–15 °C. Notably, the magnitude of temperature elevation was identical between the warmer and heatwave treatments; the only divergent factor was the duration of the thermal stress exposure. Our 10-day constant warmer treatment yielded positive growth responses for *S. japonica*, implying that this kelp can tolerate short 5-day heatwave events featuring a +∆4 °C thermal increment. The stable growth rates observed under heatwave exposure at 8 °C further support this inference. Previous studies have documented severe growth suppression in multiple kelp species subjected to prolonged acute or chronic heat stress, such as *S. japonica* [18,51] and *Macrocystis pyrifera* [52].

There was a difference in the sensitivity and acclimation to heatwaves between the two background temperature treatments. Previous physiological studies have documented that macroalgae acclimated to warm habitats generally acquire elevated heat resistance relative to conspecifics cultivated under cooler environments [34,53]. However, this study observed an opposite response in *S. japonica*, wherein marine heatwave exposure significantly inhibited sporophyte growth under the 12 °C condition, demonstrating that short-term pre-acclimation to moderate warming fails to confer enhanced tolerance against acute thermal stress. While chronic residence in warm native habitats facilitates the evolution of thermal resilience in seaweeds, such conclusions are mainly drawn from geographically separated populations with genetically encoded high-temperature adaptability. For instance, Liu and Pang (2010) [17] documented stronger photosynthetic performance under heat stress in southern *S. japonica* populations compared to their northern counterparts. Accordingly, we hypothesize that the acute heat resistance of juvenile sporophytes depends on two key factors: habitat seawater temperature and the duration of warm pre-acclimation.

Furthermore, because most macroalgae can tolerate a broad range of temperatures, growth would increase with rising temperature until the optimal temperature was achieved, at which point the growth rates would drop [54,55]. Recent work identified 13 °C as the optimal growth temperature for *S. japonica*, while temperatures exceeding 15 °C severely inhibit its growth [18,51,56]. Thus, the growth depression induced by heatwaves under warmer conditions may result from thermal exposure exceeding this species-specific optimal temperature threshold.

### 4.2. Impacts of MHWs on Young S. japonica Physiology Under Different Background Temperatures

In this study, a consistent increasing trend between RGR and pigment (Chl *c* and fucoxanthin) content was discovered under warmer conditions at the end of the heatwave and/or recovery period. In macroalgae, Chl *c* and fucoxanthin not only function as major light-harvesting pigments but also serve as critical photoprotective and antioxidant substances that mitigate thermal-induced oxidative stress [57,58]. Consistently, previous studies have demonstrated that the strategic regulation of photosynthetic pigment (Chl *a*) and accessory photoprotective pigments constitutes a key acclimation mechanism enabling seaweeds to cope with thermal stress within their physiological tolerance range [58,59,60]. In agreement with this framework, the warm 12 °C condition employed in this study significantly suppressed chlorophyll fluorescence parameters (YII, rETR, NPQ, and qP) while leaving Chl *a* content statistically unchanged. This response indicates that the 12 °C background temperature already imposes physiological pressure on the photosynthetic apparatus of juvenile *S. japonica*, and the concomitant accumulation of accessory pigments likely represents an active antioxidant strategy to counteract thermal stress. Furthermore, the pronounced decline in multiple fluorescence variables suggests that the photosystem II (PSII) reaction center is the primary target of thermal damage in heat-exposed sporophytes. Such impairment is plausibly caused by excessive ROS accumulation, which preferentially disrupts the oxygen-evolving complex (OEC) of PSII—the most thermally sensitive component of the photosynthetic machinery [19,61]. Future investigations are required to further validate this mechanistic hypothesis.

Notably, marine heatwaves exerted divergent effects on pigment accumulation in *S. japonica*, with beneficial impacts observed under the control temperature and detrimental effects detected under the warmer treatment. Compared to control treatment, the elevated levels of Chl *c* and fucoxanthin under CHW conditions indicate that a 4 °C thermal increment is sufficient to trigger positive physiological acclimation responses in this kelp. Such beneficial effects were further strengthened with prolonged thermal exposure time, as higher concentrations of Chl *c* and fucoxanthin were maintained at the heatwave and recovery stages under warmer conditions relative to the control heatwave treatment. Nevertheless, the same 4 °C heatwave perturbation induced adverse stress responses under warmer background temperatures, as evidenced by the pronounced decline in pigment synthesis. Pigment loss is a well-documented stress adaptation in macroalgae exposed to suboptimal environmental conditions, and heat-driven pigment impairment is primarily attributed to two mechanistic pathways. First, excess ROS accumulation under thermal stress directly damages thylakoid membrane integrity, triggering pigment degradation and reducing the abundance and structural stability of photosynthetic units [62,63,64]. The significantly upregulated total antioxidant capacity (T-AOC) observed in the warm heatwave treatment further supports this scenario, implying that heat-induced ROS overaccumulation activates antioxidant scavenging systems to alleviate thermal oxidative damage [62,65]. Second, thermal stress constrains nitrogen uptake and assimilation efficiency while accelerating metabolic rates, which aggravates the consumption of endogenous nitrogen reserves [32,66,67]. The reduced soluble protein (SP) content under warm heatwave conditions corroborates impaired nitrogen acquisition and assimilation under high-temperature stress, which ultimately restricts the biosynthesis of nitrogen-containing photosynthetic pigments and related functional compounds.

### 4.3. Recovery of Young S. japonica at the End of the MHW Recovery Period

Despite the significant growth inhibition and physiological disruption triggered by MHWs in warmer conditions, the adverse impacts of thermal stress were substantially alleviated during the recovery phase, reflecting the robust self-repairing capability of young *S. japonica* sporophytes. This was likely because the disruption in growth metabolism induced by oxidative pressure was offset when the temperature returned to normal during the recovery phase. As mentioned earlier, the significant upregulation of T-AOC during MHW exposure indicated prompt activation of algal antioxidant defense systems to eliminate excess ROS and repair damaged photosynthetic machinery, laying a physiological foundation for post-stress recovery. Beyond antioxidant regulation, macroalgal growth resilience is tightly coupled with nutrient uptake and assimilation capacity, and a close correlation between nutrient absorption efficiency and growth performance under favorable thermal conditions has been widely validated in various kelp species [68,69]. Soluble proteins, as vital nitrogenous compounds, provide essential amino acids and nitrogen resources for algal growth, while maintaining basic physiological stability under stress conditions [70]. In this study, soluble protein levels showed no significant difference between the warmer and MHW treatments at the end of the recovery period, demonstrating that nitrogen uptake and assimilation functions may be effectively restored after stress relief, which stabilized the growth rate of heatwave-exposed kelp. Consistent with the recovery of nitrogen metabolic homeostasis, the MHW-induced depletion of light-harvesting pigments (especially Chl *a*) was also markedly mitigated. This recovery in pigment biosynthesis is most likely driven by restored nitrogen acquisition, which reallocates available nitrogen resources to rebuild functional photosynthetic pigments. Furthermore, fucoxanthin—the key photoprotective carotenoid specific to brown macroalgae—plays a critical role in suppressing ROS overproduction in chloroplast. The increased fucoxanthin accumulation during the recovery phase further facilitates antioxidant metabolism by scavenging residual ROS, efficiently alleviating lingering thermal oxidative damage. Given that pigment content directly reflects the abundance and structural integrity of photosynthetic units [13], the recovery of pigment levels implies the restoration of the photosynthetic apparatus, which improves overall photosynthetic efficiency and sustains adequate energy supply for growth metabolism. Collectively, these lines of evidence demonstrate that the modulated synthesis of photosynthetic and photoprotective pigments serves as a core physiological regulatory strategy enabling young *S. japonica* to recover from marine heatwave stress. However, future research exploring the correlation between growth performance and nutrient uptake-allocation patterns is needed, which will advance our comprehension of how this kelp adapts to future marine environmental perturbations.

### 4.4. Ecological and Farming Implications

Habitat thermal regimes and species-specific thermal tolerance are decisive factors shaping the geographical distribution and growth phenology of seaweeds [31]. Extreme thermal events severely threaten the biomass production and physiological quality of macroalgae, particularly for cold-temperate species with narrow thermal tolerance limits [14,71,72]. In this study, young *S. japonica* cultivated under the warmer (12 °C) treatment exhibited superior growth performance and higher contents of key biochemical components (photosynthetic pigments and soluble proteins), confirming that moderate warming (12 °C) is conducive to the vegetative growth of young sporophytes. Nevertheless, the weakened resistance of warm-acclimated kelp to subsequent marine heatwaves indicates that moderately elevated background temperatures may substantially increase the risk of growth inhibition and biomass loss when acute thermal perturbations occur. This is likely because heatwaves superimposed on warmer backgrounds surpass the optimal temperature range for *S. japonica* growth, resulting in growth depression. In addition to commercial value, *S. japonica* aquaculture contributes critically to coastal ecological functions, especially blue carbon sequestration [15]. The pronounced reductions in YII, rETR, NPQ, and qP detected under both constant warming and marine heatwave treatments demonstrated that elevated temperatures impair PSII functionality and suppress the overall photosynthetic capacity of *S. japonica*. Under the progressive trend of global ocean warming, the synergistic suppression of PSII activity and vegetative growth induced by recurrent acute heatwaves may weaken the carbon fixation efficiency of *S. japonica*. Such thermal-derived physiological impairment could ultimately diminish the carbon sequestration potential of coastal kelp farms and compromise their capacity to mitigate oceanic carbon emissions in future climate scenarios.

## 5. Conclusions

In summary, this study highlights the essential role of background habitat temperature in modulating the physiological and growth responses of young *S. japonica* sporophytes to acute marine heatwave events. Our results clearly demonstrate divergent response patterns of kelp to MHWs under distinct background temperatures. Warmer seawater condition of 12 °C aided the growth and biochemical composition (pigments and soluble protein) synthesis of young *S. japonica*, whereas heatwaves mitigated and even reversed the benefits of warmer acclimation. In contrast, they had no effect at a habitat site temperature of 8 °C. Furthermore, the negative effects of MHWs on growth and biochemical composition (soluble and photosynthetic pigments) under warming conditions were alleviated after the recovery period. These results suggest that while this species can be grown in moderately warmer environments, it is not suitable for locations where acute high-temperature events take place frequently. However, the algae showed some recovery capacity when they experienced a short-period thermal stress. Given the importance of the experimental timescale on macroalgal responses, long-term heat acclimation experiments are required in the future to provide a more realistic assessment of their photosynthetic responses.

## Data Availability

The raw data supporting the conclusions of this article will be made available by the authors upon request.
